# Pancreatic Pseudocyst Fistulization: An Unusual Case of Upper GI Bleeding

**DOI:** 10.7759/cureus.12933

**Published:** 2021-01-27

**Authors:** Steve Qian, Mohamad Mouchli

**Affiliations:** 1 Internal Medicine, University of Florida College of Medicine, Gainesville, USA; 2 Gastroenterology and Hepatology, Cleveland Clinic Foundation, Cleveland, USA

**Keywords:** pancreatitis, pancreatic pseudocyst, fistula, gastrointestinal bleeding

## Abstract

The pancreas is an unusual source of gastrointestinal (GI) bleeding. GI bleeding from the pancreas is most frequently a result of complications from acute or chronic pancreatitis resulting in vascular damage and bleeding into the pancreatic duct. Rarely, however, a pancreatic pseudocyst can come into contact with the GI tract and form a fistula. However, these fistulas can be difficult to identify during endoscopy due to their lateral position, and computed tomography is often necessary to make a definitive diagnosis. Erosion of the nearby vasculature as a result of the fistula can lead to bleeding. Embolization of the affected vessel is the standard of care, but particular attention should be given to not empirically embolize due to risk of complications. Here, we describe a case of an upper GI bleed due to a pancreatic pseudocyst that fistulized through the duodenal wall.

## Introduction

The pancreas is a rare source of hemorrhage in patients with obscure gastrointestinal (GI) bleeding. Most commonly, this occurs from hemosuccus pancreaticus, which involves intrapancreatic vascular erosion, rupture, and subsequent bleeding into the pancreatic duct. However, in cases of acute pancreatitis and particularly necrotizing pancreatitis, enzymatic erosion of the duodenal wall can lead to fistulous connections between the GI tract and the pancreas. In this case report, we present the case of a patient with obscure GI bleeding (OGIB) with no signs of intraductal bleeding, instead, with bleeding from a pancreatic pseudocyst that had formed a fistula through the duodenal wall.

## Case presentation

A 65-year-old woman with past medical history significant for hypothyroidism, diabetes mellitus, hyperlipidemia, peptic ulcer disease, and colon polyps presented to the emergency room for a syncopal episode and bloody stool. Her symptoms began as left lower abdominal pain followed by an episode of stool incontinence and bright red blood per rectum with large clots. The patient additionally endorsed a four-week history of poor appetite and intermittent left lower abdominal pain. She reported chronic use of non-steroidal anti-inflammatory drugs for joint pain. Of note, she was hospitalized two months prior at another institution for pancreatitis with complete recovery after a few days. Workup at the time did not reveal gallstones, and the patient denied a history of alcohol abuse.

Vital signs were stable on presentation. Initial physical examination revealed dried blood in the rectum, but was otherwise unremarkable. There was no abdominal tenderness. Laboratory study results were significant for a hemoglobin of 5.9 g/dL, white cell count of 25,000/μL, creatinine of 1.72 mg/dL, urea nitrogen of 30 mg/dL, and lipase of 345 mmol/L. She was transfused two units of packed cells and started on acid-suppression therapy and intravenous fluids. Blood and urine cultures were negative and her leukocytosis improved with hydration. Abdominal imaging was not obtained at the time of admission due to presence of an acute kidney injury.

**Figure 1:**
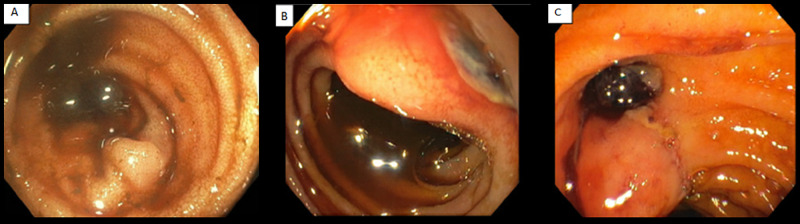
(a) Colonoscopy revealing old blood in the colon, (b) push enteroscopy showing a lateral ulcer, and (c) duodenoscopy confirming a deep penetrating ulcer in the second portion of the duodenum.

Esophagogastroduodenoscopy (EGD) and colonoscopy were unremarkable aside from old blood seen throughout the colon and terminal ileum (Figure 1a). Follow-up capsule endoscopy revealed old blood in the distal duodenum, proximal jejunum, and distal ileum, increasing suspicion for small bowel bleeding. A follow-up EGD with push enteroscopy was initially negative, but as the patient became increasingly unstable, the procedure was repeated. Upon closer inspection, we identified what appeared to be a small, hidden, deeply penetrating ulcer between folds in the second portion of the duodenum (Figure 1b). During the procedure, the duodenoscope was introduced and confirmed the ulcer was above the minor papillary fold (Figure 1c). The patient subsequently underwent urgent angiography which did not reveal active hemorrhage. However, as the patient continued to have signs of bleeding, the gastroduodenal artery (GDA) was embolized during the procedure. Soon after, however, the patient rapidly deteriorated into hemorrhagic shock requiring massive blood transfusion.

**Figure 2:**
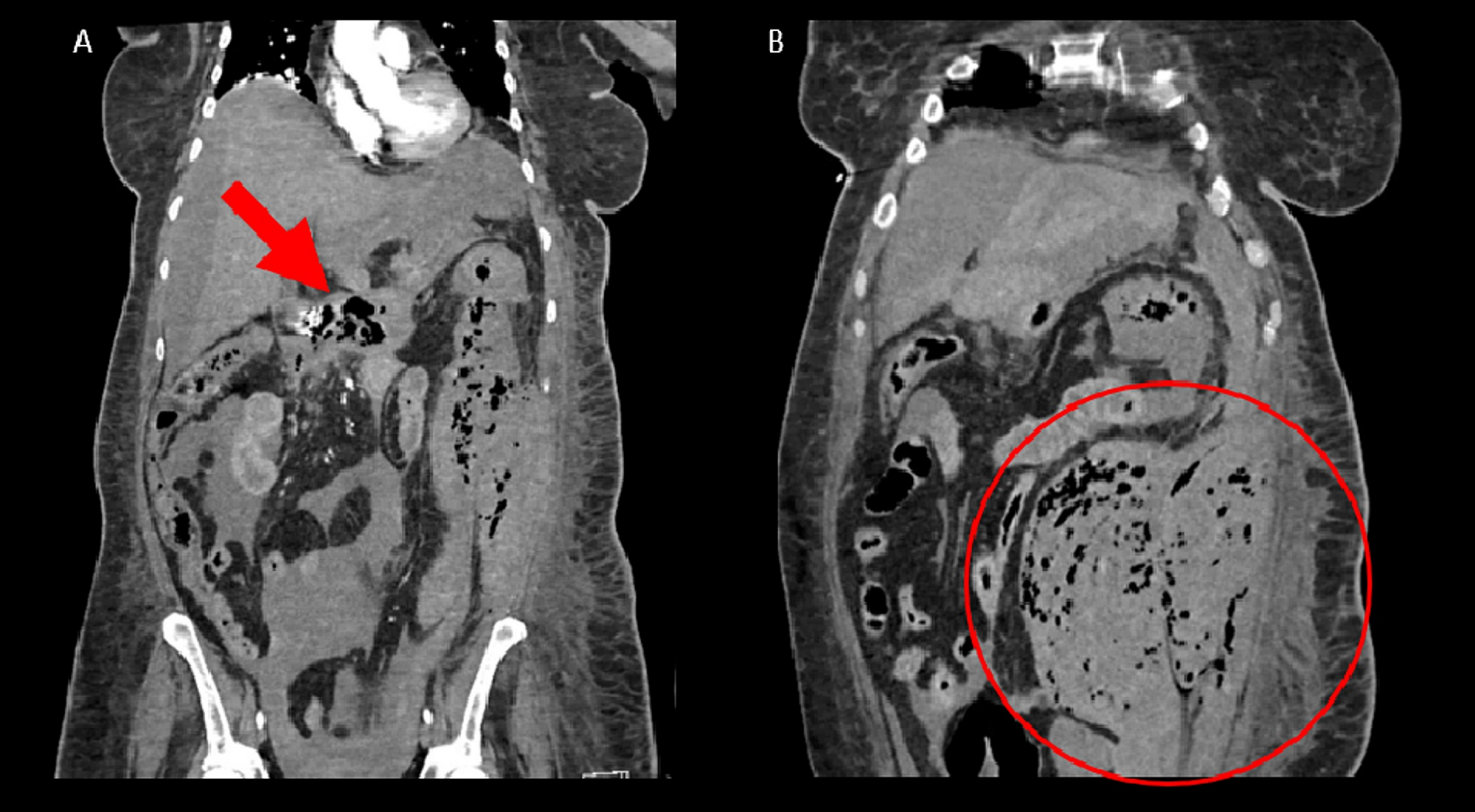
Coronal views of CT angiography revealing (a) emphysematous pancreatitis with (b) hemoperitoneum. CT, computed tomography

A computed tomography (CT) angiogram of the abdomen and pelvis was obtained after embolization, which revealed hemoperitoneum, severe emphysematous pancreatitis with active hemorrhage, possible splenic artery aneurysm, and scattered air throughout the abdomen and pelvis (Figures 2a, 2b). The patient developed abdominal compartment syndrome and was taken to the operating room for splenic artery coil embolization, retroperitoneal exploration, decompressive laparotomy, necrosectomy, and partial omentectomy. She remained critically ill in the intensive care unit on multiple pressors and required numerous surgical procedures for complications, but continued to deteriorate. After two weeks in intensive care, the family elected to proceed with comfort care.

## Discussion

OGIB is defined as GI bleeding with a source that cannot be identified by EGD and colonoscopy. Rarely, the bleeding can be of extraluminal origin such as from the biliary tract, the pancreas, or an aortoenteric fistula [1]. In the pancreas, the most common cause of OGIB is hemosuccus pancreaticus [1]. This is typically due to rupture of a pseudoaneurysm, most commonly of the splenic artery [2], into the pancreatic duct.

In our case, EGD and duodenoscopy revealed what initially appeared to be an ulcer, but turned out to be a fistula from a pancreatic pseudocyst on further imaging. This pseudocyst likely formed from the patient’s prior episode of pancreatitis, although this could not be confirmed due to a lack of a baseline CT scan. The pseudocyst eroded into an artery in the wall of the duodenum, leading to bleeding from the site. During angiography, empiric embolization of the GDA was performed to stem any residual bleeding. However, afterwards, the patient began to decompensate from massive hemorrhagic shock. Embolization of the GDA would have resulted in a redirection of the blood flow into the splenic vasculature, potentially generating new sites of bleeding given the patient’s active emphysematous pancreatitis. This was evident on CT imaging, which revealed hemoperitoneum and severe pancreatic bleeding.

Pancreatic pseudocysts can lead to fistula formation in the GI tract, including the stomach, duodenum, and colon, or to nearby vascular structures such as the splenic or portal veins [3]. The etiology of this process includes release of pancreatic enzymes from the pseudocyst and local ischemia from compression of the pseudocyst [4]. Fistulization to the GI tract results in hemorrhage, presenting as hematemesis, hematochezia, melena, or intra-abdominal bleeding [3]. The bleeding is typically acute in nature, but can on occasion present as slow intermittent bleeding. Fistulization to vascular structures presents with signs of portal hypertension, venous congestion, or rarely, mesenteric steal syndrome [5,6].

Diagnosis of pancreatic pseudocyst fistulization can be challenging given the rarity of the condition, with a reported incidence of 6-10% in cases of chronic pancreatitis [7]. When there is high clinical suspicion, early CT imaging is critical in making a rapid diagnosis [8], but was not feasible here due to acute kidney injury. Endoscopy may be able to identify the site of fistulization, although this can be difficult if the bleeding site is lateral, as in this case [9]. If active bleeding is identified, the patient should immediately undergo catheter embolization of the affected vessel with surgical repair of the fistula for definitive treatment [10]. If a bleeding site is not identified, the patient should not undergo empiric embolization if there is active pancreatitis due to the possibility of worsening bleeding, as in this case.

## Conclusions

Pancreatic pseudocyst fistulization is a cause of OGIB that should be considered in patients who present with GI bleeding of unclear etiology with signs and symptoms of acute pancreatitis or a history of chronic pancreatitis. Patients should be diagnosed with prompt CT angiography, particularly if bidirectional endoscopy does not identify a cause of bleeding. Management should involve embolization of the affected artery and surgical repair if a source of bleeding is identified. If no source is identified, empiric embolization should be avoided, especially if there is presence of acute pancreatitis. This case highlights an unusual presentation of OGIB, resulting from fistulization of a pancreatic pseudocyst through the wall of the duodenum.
